# Submerged Spark Plasma Discharges for the Removal of Antibiotics, Antibiotic-Resistant Bacteria, and Resistance Genes

**DOI:** 10.1007/s11090-026-10705-0

**Published:** 2026-08-20

**Authors:** Jinjie He, Gregory Fridman, Charles Bailey, Alexander Rabinovich, Alexander Fridman, Christopher M. Sales

**Affiliations:** 1 C. & J. Nyheim Plasma Institute, Drexel University, Camden, NJ USA; 2 Civil, Architectural, and Environmental Engineering, Drexel University, Philadelphia, PA USA; 3 AAPlasma LLC, Grove City, OH USA

**Keywords:** Spark plasma, Antibiotics, Antibiotic resistance bacteria, Antibiotic resistance genes, Water treatment

## Abstract

The widespread use of antibiotics in aquaculture and livestock production has contributed to the dissemination of antibiotics, antibiotic-resistant bacteria (ARB), and antibiotic resistance genes (ARGs) in aquatic environments. In this study, submerged spark plasma systems were evaluated for the removal of rifampicin, rifampicin-resistant *Escherichia coli* O157:H7, and ARG-associated DNA in different water matrices using a 40 mL single-spark reactor and a 1000 mL multiple-spark reactor. The single-spark system achieved 99.43% removal of rifampicin within 8 min and >5-log reduction of *E. coli* O157:H7 within 10 min in distilled water. The multiple-spark system achieved up to 78.46% rifampicin removal and >5-log bacterial inactivation depending on power input. Plasma treatment also reduced ARG-associated DNA, including up to 4-log reduction in total DNA and rapid degradation of extracellular DNA to below detection limits within 1 min. Rifampicin degradation followed pseudo-first-order kinetics in the single-spark reactor and pseudo-second-order kinetics in the multiple-spark reactor. Saline water conditions significantly reduced antibiotic degradation, bacterial inactivation, and ARG reduction efficiencies. The lowest electrical energy per order (E_EO_) for *E. coli* inactivation was 1.2 kWhr/m^3^ per 1-log reduction at 60 W in the multiple-spark system. Compared to sodium hypochlorite treatment, submerged spark plasma achieved greater rifampicin degradation and faster extracellular DNA reduction without chemical additives. These findings demonstrate the potential of submerged spark plasma as a multifunctional advanced oxidation and disinfection technology for mitigation of AMR-related contaminants in water treatment applications.

## Introduction

The growing global demand for animal protein has led to the expansion of livestock farming and aquaculture. While intensive production improves yield, it also elevates the risk of infectious diseases [1]. Antibiotics are therefore widely used in animal husbandry as they have the ability to kill or inhibit the growth of pathogens [2]. However, the overuse and misuse of antibiotics have contributed to the emergence and dissemination of antibiotic resistance, including antibiotic-resistant bacteria (ARB) and antibiotic resistance genes (ARGs). Both ARGs and antibiotics are now recognized as emerging pollutants worldwide [3]. ARGs can be transferred among microorganisms, potentially spreading antibiotic resistance to different bacterial strains [4]. This poses a serious threat to human health, as it can lead to reduced treatment efficacy, increased toxicity, and the potential for allergic reactions [5]. The discharge of aquaculture effluents containing residual antibiotics, ARB, and ARGs further amplifies antimicrobial resistance (AMR) risks, pointing to the critical need for effective treatment approaches.

Conventional water treatment and disinfection technologies have shown varying effectiveness for the removal of antibiotics, ARB, and ARGs. Chlorination and UV treatment can effectively inactivate bacteria but often fail to adequately remove ARGs [6, 7]. Membrane bioreactor (MBR) systems have the capability to completely remove ARB, a portion of ARGs, and antibiotics from wastewater, but the high maintenance costs associated with MBR limit application [8]. Advanced oxidation processes (AOP), such as Fenton, photo-Fenton, photolysis, ozonation and electrochemical oxidation, are demonstrated to be effective to reduce the antibiotics [9, 10]. Nevertheless, these methods may require high dosages, external additives, acidic conditions, and may generate disinfection by-products (DBPs), raising concerns about potential human health risks. For instance, while ozone at high doses can reduce ARGs in water, regular doses used for disinfection do not eliminate intracellular ARGs [11]. Moreover, certain disinfection methods like photo-Fenton, UV, and ozone may induce apoptosis in bacteria, leading to the release of bacterial DNA and an increase in ARGs [12, 13].

Non-thermal plasma has emerged as a promising advanced oxidation and disinfection technology for water treatment applications. Plasma discharges generated underwater can produce a complex mixture of reactive oxygen and nitrogen species (ROS/RNS), UV, transient electric fields, shockwaves, and localized high-temperature microchannels capable of degrading organic contaminants and inactivating microorganisms [14–23]. Among various plasma configurations, underwater spark plasma is particularly attractive because it enables efficient plasma–liquid interactions and in situ formation of reactive species. Previous studies have demonstrated the effectiveness of submerged plasma systems for degradation of organic pollutants [24] and microbial inactivation [25]. However, many existing studies have focused primarily on either chemical contaminant degradation or microbial inactivation independently, while comparatively few investigations have evaluated the simultaneous removal of antibiotics, ARB, and ARG-associated DNA within the same treatment system.

In this study, the performance of submerged spark plasma was comprehensively evaluated for the simultaneous removal of rifampicin, rifampicin-resistant *Escherichia coli*, and associated ARGs. The spark discharges were generated within air bubbles introduced near submerged electrodes, allowing plasma formation at the gas–liquid interface while maintaining direct interaction with the surrounding liquid phase. Two reactor configurations, single-spark and multiple-spark systems, were investigated to assess the influence of power input, treatment volume, and scalability on process performance. In addition to degradation and inactivation efficiency, kinetic behavior and specific energy consumption were analyzed to identify optimal operating conditions. The applicability of the system was further examined across different water matrices, including distilled water, freshwater, and saline water, to assess its potential for aquaculture applications. A comparison with sodium hypochlorite (NaClO) was also conducted to benchmark plasma performance against a conventional disinfectant.

## Materials and Methods

### Preparation of *E. coli* and Viable Bacteria Quantification

The Rifampicin-resistant strain of *Escherichia coli* O157:H7 (ATCC 700728) was kindly provided by Dr. N. Nitin at University of California at Davis, Davis, CA, USA. Fresh culture of strain O157:H7 was prepared in Tryptone Soy Broth (Becton Dickinson, USA) containing 100 μg/mL of rifampicin (Sigma Aldrich, USA) at 37 °C overnight. The final bacteria concentration in colony forming units per mL (CFU/mL) was confirmed by plating on tryptone soy agar (TSA) containing 20 μg/mL rifampicin. Once water samples were collected, serial dilutions were made with sterile phosphate buffered saline (PBS). Then 500 μL of appropriate dilution was spread uniformly on rifampicin contained TSA agar plates and incubated at 37 °C overnight for CFU counting.

### Plasma Treatment

**Fig. 1:**
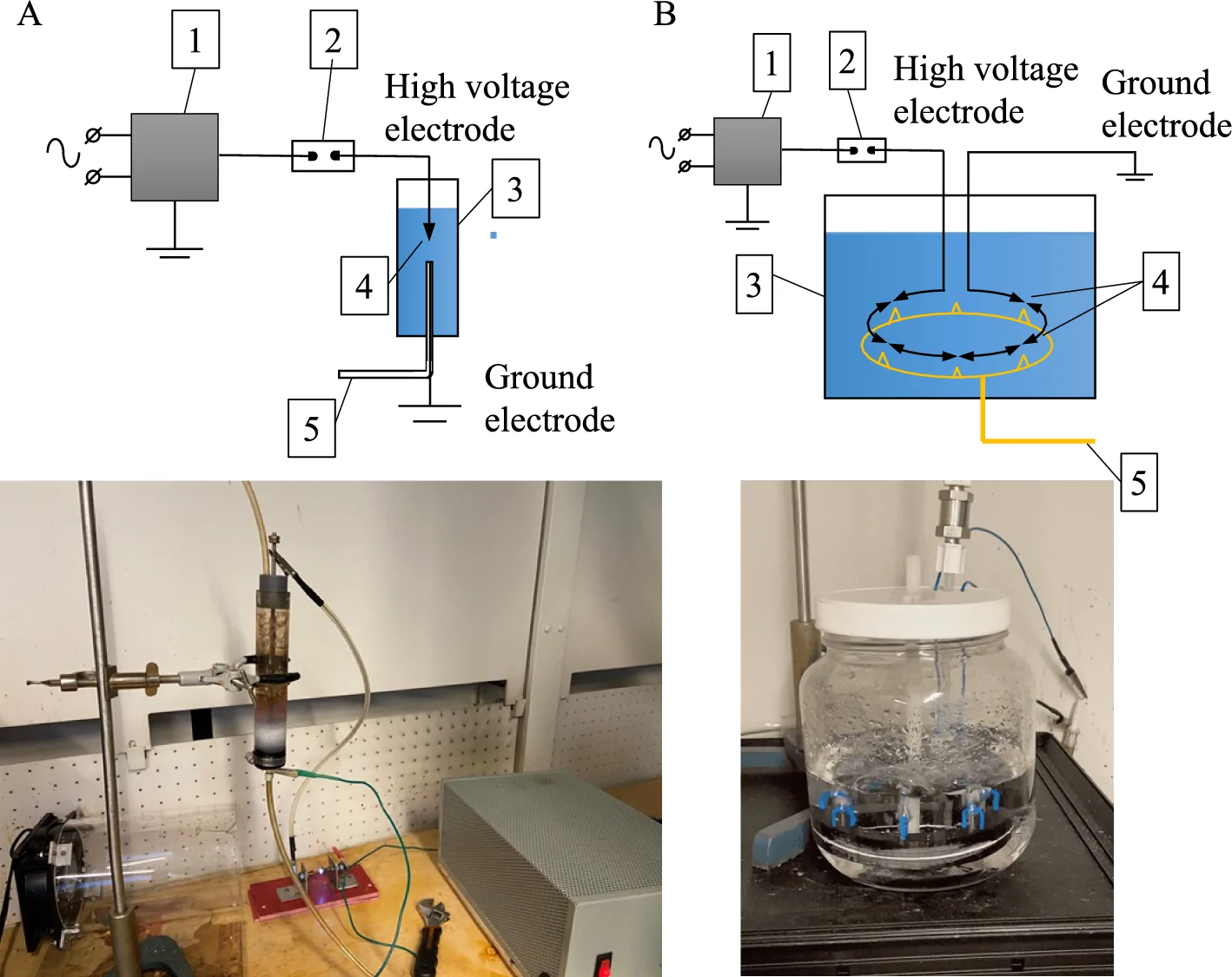
Electrical diagram of two plasma systems. **A** is the 40 mL single-spark plasma system in tube, **B** is the 1000 mL multiple-spark plasma system in jar. 1 – 10kV DC power supply; 2 – Adjustable air gap switch; 3 – Sparks discharge reactor; 4 – Spark gap; 5 – Air bubbler line

Two submerged spark plasma systems were evaluated in this study, including (a) a 40 mL single-spark plasma system, and (b) a 1000 mL multiple-spark plasma system (Fig. 1). In both systems, plasma discharges were generated between 316 stainless steel electrodes connected to a 10–12 kV DC power supply through an adjustable high-voltage air-gap switch. The switching air gap was maintained at approximately 5–7 mm to generate repetitive spark discharges. The spark was generated between high voltage electrode and ground electrode that were submerged in the water inside the reactor. Compressed Air was directed to the plasma discharge by air bubble line at a flow rate of approximately 3–5 L/min. The discharge frequency was approximately 18–20 Hz. Individual spark discharges had a duration of approximately 2–3 μs. The input power of single-spark plasma system was 100–120 W, while the input power of multiple-spark system was 25–75 W.

The single-spark reactor operated with a treatment volume of 40 mL and relatively high local energy density, while the multiple-spark system treated volumes up to 800–1000 mL with distributed discharge behavior. Freshly cultured *E. coli* or rifampicin was intentionally added to different types of water samples, including distilled water, tap water, 3.5% salt water, and aquaculture water. Subsequently, the solution was subjected to treatment in a plasma unit for varying durations. Following the treatment, water samples were extracted from the reactor to quantify the presence of *E. coli* or analyze the levels of rifampicin. The 3.5% salt water was prepared by adding 35 g NaCl to 1 L of distilled water to mimic the salt concentration of seawater.

### DNA Extraction and Quantitative Polymerase Chain Reaction (qPCR) Analysis

In this study, water samples were collected and processed to extract three types of DNA: total DNA, intracellular DNA, and extracellular DNA. The goal was to quantify the abundance of *E. coli* antibiotic resistance genes using qPCR analyses. Total DNA was directly extracted from the water samples themselves. Extracellular DNA, on the other hand, was extracted from the water that had been filtered using a 0.22 μm filter unit. The intracellular DNA, however, was extracted specifically from the filter paper used in the filtration process. The DNA was extracted by using Qiagen QIAmp Fast DNA Stool Mini Kit following the manufacturer’s instructions [26]. The concentration of the extracted DNA for each sample was measured spectrophotometrically via NanoDrop and fluorimetrically via QuBit 2.0.

Primer sets are targeting a gene on the chromosome encoding for protein MarA, which is involved in reducing permeability and increasing antibiotic efflux of *E.coli* O157:H7 (E.coli_RP_F 5'-TAAATGGCACCTGCAACGGA-3'; E.coli_RP_R 5'-GTCATCTTACGGCTGCGGAT-3') were designed and ordered from IDT DNA. A fast SYBR Green qPCR assay was applied to obtain the concentration of the DNA extracted water samples. All samples were stored at −20 °C until they were analyzed.

A QuantStudio 3 Real-Time PCR System and Applied Biosystems Fast SYBR Green Master Mix were used to conduct all qPCR assays. Total qPCR reaction volume was 20 μL. Each reaction mixture contained 6 μM of the forward and reverse primers, 2 μL of template DNA and 10 μL of fast SYBR Green Master Mix. The program employed: pre-incubation for 20 s at 95 °C; 40 amplification cycles of 1 s of denaturing at 95 °C and 20 s of annealing at 60 °C; and, finally, 1 s of 95 °C, 20 s of 60 °C and 1 s of 95 °C for melt curve. All assays were conducted in triplicates of each sample with negative controls (no template) and positive controls.

### Rifampicin Quantification

Rifampicin is widely used in clinical practice and has been found in a number of aquatic products [27]. In this study, the concentration of rifampicin was determined using UV–visible spectrophotometry (UV–vis) or high-performance liquid chromatography (HPLC) techniques. To establish a calibration curve, standard solutions containing known amounts of rifampicin in phosphate buffer pH 7.4 were prepared. For UV–vis analysis, measurements were taken at wavelengths of 332 nm and 475 nm. The concentration of rifampicin was determined from a calibration curve based on the summed absorbance values at both wavelengths.

For HPLC analysis, a C-18 column (3 µm, 120 A, 2.1 × 150 mm) was utilized. The mobile phase consisted of a mixture of methanol and potassium phosphate buffer (0.02 M, pH 7) in a ratio of 3:1, v/v. The mobile phase was pumped through the column at a flow rate of 0.5 mL/min. The temperature of the system was maintained at 30 °C, and the injection loop had a volume of 10 µL. The detector was set at a wavelength of 339 nm to detect rifampicin. Each sample was measured three times, and the mean value was calculated to ensure accuracy.

### Aquaculture Water Collection

Aquaculture fresh water was collected from a shellfish culture tank in Delaware State University Aquaculture Research and Demonstration Facility (see Acknowledgements). Samples were stored in 1 L polyethylene bottles and transported on ice to the laboratory immediately after collection (within 6 h) and keep refrigerated at 4 °C until test in the next day of collection.

### Calculations of Removal Efficiencies

Removal efficiency of antibiotic residues was evaluated in the relative removal efficiency (%), which was calculated in Eq. 1 as follows:

1 $$Removal Efficiency \left(\%\right)=\left(\frac{{{C}_{\mathrm{u}\mathrm{n}\mathrm{t}\mathrm{r}\mathrm{e}\mathrm{a}\mathrm{t}\mathrm{e}\mathrm{d}}-C}_{\mathrm{t}\mathrm{r}\mathrm{e}\mathrm{a}\mathrm{t}\mathrm{e}\mathrm{d}}}{{C}_{\mathrm{u}\mathrm{n}\mathrm{t}\mathrm{r}\mathrm{e}\mathrm{a}\mathrm{t}\mathrm{e}\mathrm{d}}}\right)\times 100\%$$

Removal efficiency of ARB and ARGs were measured in log reduction:

2 $$Log Reduction ={log}_{10} \left(\frac{{C}_{\mathrm{t}\mathrm{r}\mathrm{e}\mathrm{a}\mathrm{t}\mathrm{e}\mathrm{d}}}{{C}_{\mathrm{u}\mathrm{n}\mathrm{t}\mathrm{r}\mathrm{e}\mathrm{a}\mathrm{t}\mathrm{e}\mathrm{d}}}\right)$$

### Electrical Energy Per Order Evaluation

Energy efficiency was determined as electrical energy per order (*E*_*EO*_) in this study. *E*_*EO*_ can be defined as the number of kW-hr (kilo-Watt-hour) of electrical energy required to reduce the concentration of a contaminant by an order of magnitude in the concentration in a unit volume [28]. The electrical energy per order can be calculated by using the Eq. 3 [29]

3 $${E}_{EO}=\frac{P\times {t}_{x}\times {10}^{3}}{V\times 60}$$

where *P* is the rated power (in W), *t*_*x*_ is the treatment time (in min) to achieve the first magnitude reduction, *V* is the volume of effluent taken (in mL), *C*_*0*_ and *C*_*t*_ is the contaminant concentration (in ppm) at initial and final. The unit of E_EO_ is kWhr/m^3^ order 1.

The removal of rifampicin over time from experiments were fit to either zero (Eq. 4), pseudo-first (Eq. 5) or pseudo-second-order (Eq. 6) kinetic models to determine removal rate constants.

4 $${C}_{0}-{C}_{t}=kt$$

5 $$\mathrm{ln}\left(\frac{{C}_{0}}{{C}_{t}}\right)=kt$$

6 $$\frac{1}{{C}_{t}}-\frac{1}{{C}_{0}}=kt$$

For the above Eqs. (4–6), *k* is the rate constant for the decay of the rifampicin concentration. *C*_*0*_ is initial concentration of rifampicin; *C*_*t*_ is the rifampicin concentration at any time.

To simplify the equation to determine E_EO_, Eqs. 4, 5 and 6 were substituted into Eq. 3 to obtain following result for different orders of reactions. For the removal of 1-log *E. coli*, C_0_/C_t_ = 10 was substituted to the equations:

7 $${E}_{EO}=\frac{15\times P\times {C}_{0}}{V\times k} for\; zero\; order$$

8 $${E}_{EO}=\frac{38.4\times P}{V\times k} for\; first\; order$$

9 $${E}_{EO}=\frac{150\times P}{V\times k\times {C}_{0}} for \;second order$$

## Results and Discussion

### Removal of Rifampicin

**Fig. 2:**
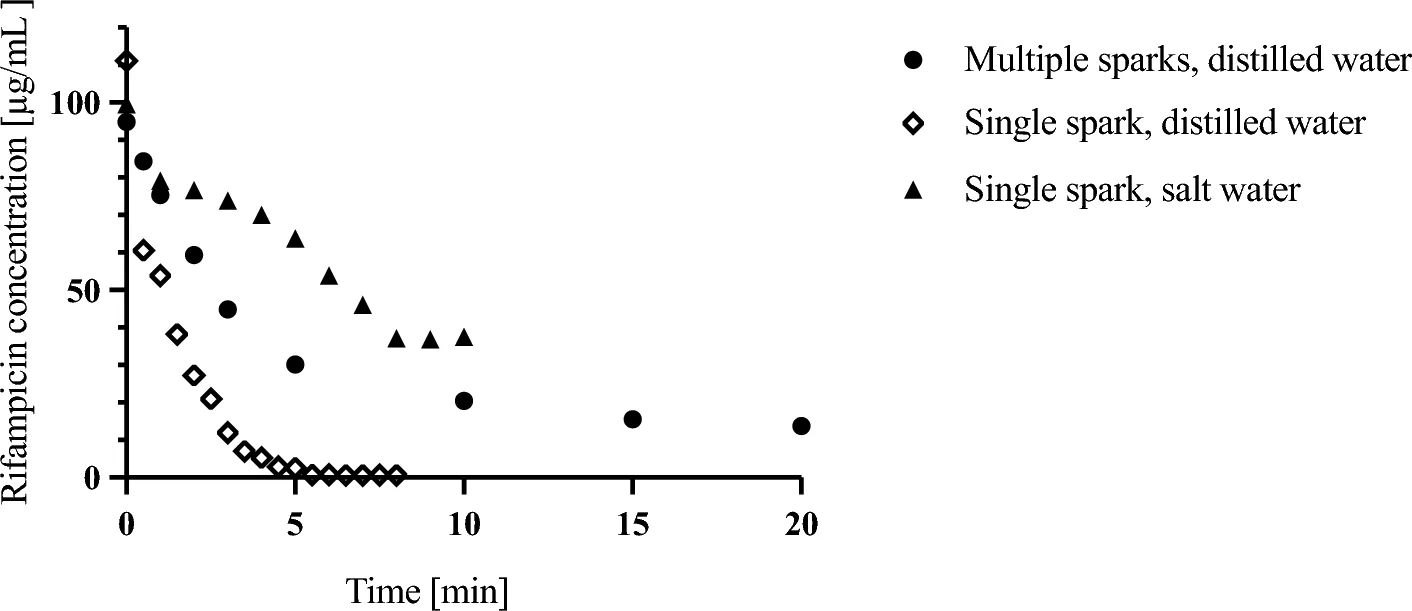
Variation of rifampicin in distilled water and 3.5% salt water treated by multiple-spark and single-spark plasma

The single-spark and multiple-spark plasma system effectively removed rifampicin from water (Fig. 2). Rifampicin was spiked in water with an initial concentration of 100 μg/mL. In the single-spark system (40 mL, 100–120W), 99.43% removal efficiency was achieved in distilled water within 8 min, whereas only 62.71% removal was observed in 3.5% saltwater over the same time frame. The multiple-spark system (800 mL, 75 W) demonstrated a removal efficiency of 78.46% in 10 min in distilled water and 85.54% in 20 min.

Kinetic analysis was conducted using zero, first, and second order kinetic models (Table 1). Degradation in the single-spark system (40 mL, 100–120 W) correlated well with a first-order kinetic model in both distilled water and saltwater, with a higher rate constant in distilled water, indicating faster degradation under low ionic strength conditions. In contrast, data from multiple-spark system (800 mL, 75 W) followed second-order kinetic model with a kinetic constant of 0.003, likely due to lower power density and larger treatment volume. Reduced energy input per unit volume may cause the reaction rate to depend on both rifampicin concentration and reactive species availability. The higher power input and smaller volume of the single-spark system likely promote a more concentrated and uniform distribution of reactive species, resulting in simplified first-order behavior.

The effectiveness of sodium hypochlorite (NaClO), which is a disinfectant widely used in aquaculture, was also evaluated. At a concentration of 50 ppm, NaClO demonstrated limited impact on rifampicin removal in both distilled water and saltwater. After 30 min of reaction, it only achieved 7.4% and 13.4% removal, respectively. These findings suggest that NaClO is not a viable option for the effective removal of rifampicin.

The energy costs for rifampicin removal using spark plasma treatment varied significantly across different systems and water matrices. In the 40 mL single-spark system, achieving a 1-log reduction in distilled water required 132 kWhr/m^3^, while in 3.5% saltwater, the cost increased to 518 kWhr/m^3^ (Table 2). In contrast, the multiple-spark system was more energy-efficient, requiring only 44 kWhr/m^3^ for a 1-log reduction in distilled water. These findings demonstrate that while spark plasma is effective in low-salinity water, further optimization is required for saline applications.

**Table 1:** The kinetics equations and parameters of zero-, first- and second order reactions of rifampicin degradation at different treatment condition

Plasma system	Fitted equation	Reaction order	k (min^−1^)	R^2^
Multiple spark-DI water	Y=−3.7112x+72.043	Zero-order	3.7112	0.7324
Single spark-DI water	Y=−9.8041x+59.553	Zero-order	9.8041	0.6639
Single spark-Salt water	Y=−7.2095x+99.600	Zero-order	7.2095	0.9496
Multiple spark-DI water	Y=−0.0981x+4.2811	First-order	0.0981	0.8898
Single spark-DI water	Y=−0.7248x+4.5796	First-order	0.7248	0.9584
Single spark-Salt water	Y=−0.1029x+4.5763	First-order	0.1029	0.9529
Multiple spark-DI water	Y=0.0033x+0.0120	Second-order	0.0033	0.9818
Single spark-DI water	Y=0.2402x-0.3663	Second-order	0.2402	0.8134
Single spark-Salt water	Y=0.0018x+0.0090	Second-order	0.0018	0.9226

**Table 2:** Energy cost of single-spark and multiple-spark system treating DI water and 3.5% salt water for rifampicin

Plasma system	Volume [mL]	Substrate	Power [Watts]	E_EO_ [kWhr/m^3^ 1-log]	Cost* [$/m^3^ 1-log]	Reaction order	Rate constant [min^−1^]	R^2^
Multiple sparks	800	Distilled water	75	44	3.13	Second order	0.003	0.981
Single spark	40	Distilled water	100–120	132–159	10.18–12.26	First order	0.725	0.958
Single spark	40	3.5% salt water	100–120	932–1118	71.86–86.20	First order	0.103	0.953

*Cost was calculated based on the Average Price of Electricity to Ultimate Customers by End-Use Sector, the price in Pennsylvania was $0.0771 per kWhr for industrial use in May 2023 [30]

### Removal of ARB

**Fig. 3:**
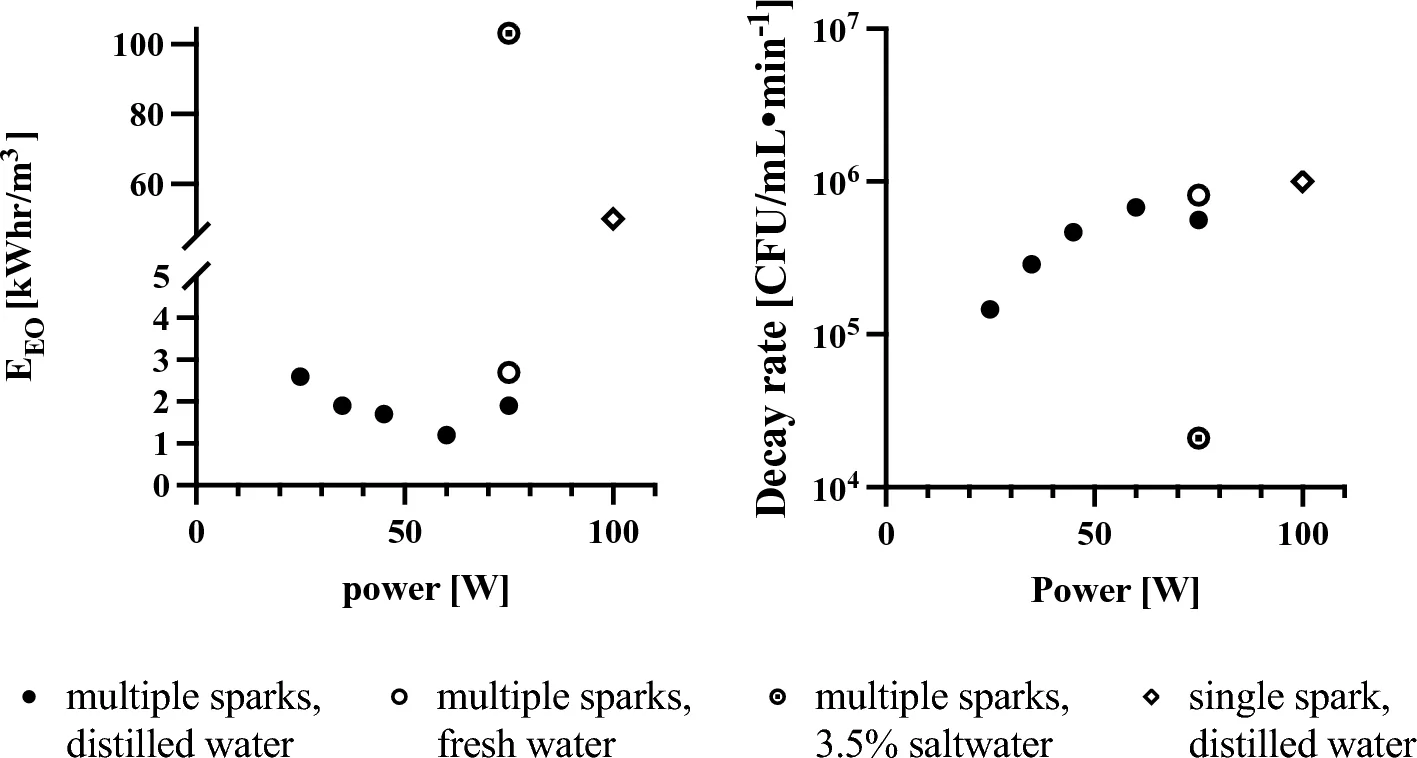
Energy cost and *E. coli* inactivation rate in distilled water, fresh water and 3.5% saltwater with the input power of 25–100 W in the single-spark and multiple-spark system

The antimicrobial efficiency of spark plasma was evaluated using *E. coli* O157:H7. In the single-spark system (100–120 W), 30 mL of distilled water containing *E. coli* at an initial concentration of 10^5^ CFU/mL was treated (Fig. 3). A 5-log reduction in *E. coli* was achieved within 10 min. The inactivation kinetics followed a second-order reaction model with R^2^ of 0.917.

The multiple-spark system was evaluated with power levels ranging from 25 to 75 W to treat 800 mL of distilled water. The inactivation kinetics in this system followed a first-order reaction with R^2^ over 0.9. At an initial concentration of 5.7 × 10^5^ CFU/mL, more than a 2-log reduction in *E. coli* was achieved within 10 min at 25 W. A 3-log reduction was obtained at 35 W, while power levels above 45 W resulted in greater than 5-log within the same 10-min treatment period. These results indicated that increasing plasma power enhances the rate of *E. coli* inactivation. However, treating 3.5% saltwater in this system proved to be challenging. In 10 min, only 0.5-log (34%) of the *E. coli* in saltwater was removed, whereas at the same power level of 75 W, 0.7-log (78%) of *E. coli* in distilled water was inactivated.

### Removal of ARGs

**Fig. 4:**
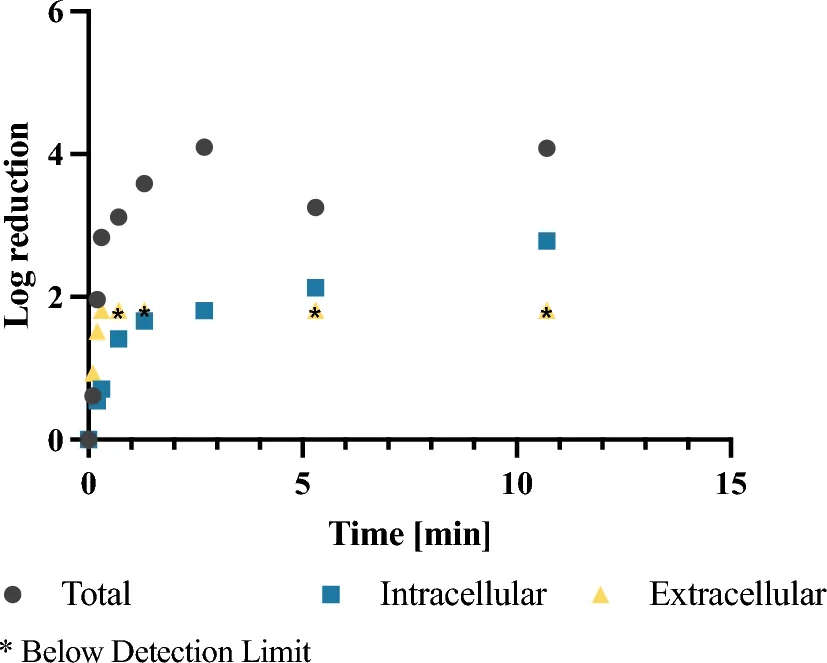
Log reduction of *E. coli* DNA including total, intracellular and extracellular DNA treated in 40 mL single-spark system over time. * Below detection limit 10,000 CN/100 mL

To evaluate the ARG removal efficacy of the spark plasma system, the DNA of *E. coli* was extracted and quantified via qPCR as an alternative measurement for ARGs. The target sequence was on the chromosome encoding for protein MarA, which is a multiple antibiotic resistance transcriptional regulator. In the single-spark system, total DNA, intracellular DNA, and extracellular DNA were measured to determine the effectiveness of spark plasma treatment in distilled water (Fig. 4). Plasma treatment achieved a 4-log reduction in total DNA and a 2.8-log reduction in intracellular DNA within 10 min. More than a 1.8-log reduction in extracellular DNA was observed within 20 s, and concentration fell below the detection limit (6068 CN/100 mL) after 1 min of treatment.

**Fig. 5:**
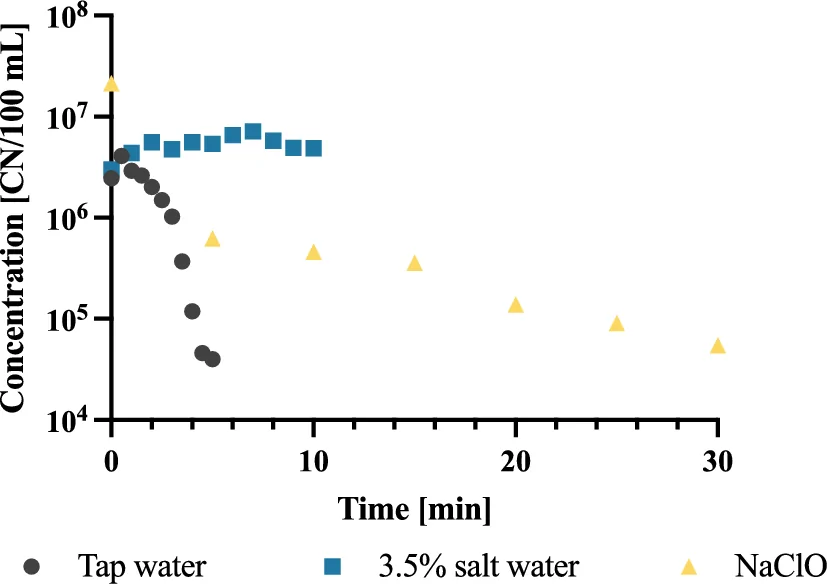
Total *E. coli* DNA treated in 800 mL tap water and 3.5% salt water by multiple-spark system and 50 ppm NaClO for various duration

In the multiple-spark system, a larger volume of water (80 mL) was treated (Fig. 5). A 1.8-log reduction in total DNA was achieved within 5 min in tap water. By comparison, 50 ppm NaClO required 15 min to achieve the same reduction. In 3.5% saltwater, no significant DNA reduction was observed, likely due to the inhibitory effect of high ionic strength on spark discharge formation and reactive species generation.

### Plasma Treatment Mechanisms

The submerged spark plasma systems evaluated in this study generated a highly reactive physicochemical environment capable of simultaneously degrading antibiotics, inactivating ARB, and reducing ARG-associated DNA. Plasma discharges formed under water produces a complex mixture of ROS and RNS, including radicals, hydrogen peroxide, singlet oxygen, superoxide anions, and ozone, nitrate, nitrite, peroxynitrite, nitric oxide radical, ammonia, and nitrogen species [31]. Compared with other non-thermal plasma sources, such as microwave plasma and dielectric barrier discharge (DBD), which are typically generated above the liquid surface, and rely primarily on gas-phase transport of reactive species into the liquid, the submerged spark facilitates rapid transfer of both short-lived and long-lived reactive species to the surrounding water. In addition to chemical oxidants, submerged spark discharges also generate UV, transient electric fields, localized high-temperature microchannels, and shockwaves. These combined physicochemical effects likely contributed synergistically to the degradation of rifampicin, bacterial inactivation, and DNA damage observed in this study [32].

Reactor configuration strongly influenced plasma behavior and treatment performance. The single-spark system operated with a relatively small treatment volume and higher local energy density, resulting in rapid rifampicin degradation and faster bacterial inactivation. Under these conditions, reactive species were likely generated at sufficiently high concentrations to maintain excess oxidant availability throughout treatment, leading to degradation kinetics that followed a pseudo-first-order model. In contrast, the multiple-spark system treated substantially larger liquid volumes at lower effective power density. The degradation behavior in this reactor followed pseudo-second-order kinetics, suggesting that contaminant removal became increasingly dependent on both contaminant concentration and reactive species availability. These observations indicate that reactor scale and discharge distribution significantly influence plasma-liquid interactions and reaction pathways.

The degradation of rifampicin is likely associated with oxidative attack by plasma-generated ROS, which (including O_2∙_^−^, ^∙^OH, and ^1^O_2_) are known to degrade antibiotics such as tetracycline (TC), sulfadiazine (SD), and ciprofloxacin (CIP) in previous research [33]. Rifampicin contains multiple susceptible functional groups, including three C–N bonds and one N–N bond. Plasma-generated ROS may preferentially attack the relatively weak N–N bond in rifampicin molecule, initiating bond cleavage followed by demethylation, dehydration, acetyl group detachment, aromatic ring oxidation, and chain scission reactions, resulting in the formation of smaller intermediate compounds [34, 35].

The inactivation of *E. coli* O157:H7 was also strongly influenced by plasma generated reactive species and physical plasma effects [14, 36, 37]. ROS and RNS can induce oxidative damage to bacterial membranes, proteins, and intracellular biomolecules, ultimately leading to cell death. Hydroxyl radicals and hydrogen peroxide are particularly important due to their high oxidation potential and ability to initiate lipid peroxidation and membrane disruption [38–44]. In addition to oxidative stress, transient electric fields generated during spark discharges may contribute to membrane destabilization and electroporation, increasing membrane permeability and cellular leakage [45–47]. Shockwaves generated by plasma channel expansion may further contribute to mechanical disruption of bacterial structures [48, 49]. Molecular dynamics simulations have shown that shockwaves can form pores on the bacterial cell membrane, leading to controlled expansion and cell damage [50]. The role of UV remains debated, with some studies suggesting negligible effects [43, 45, 48], while the others stated it could cause lethal damage of DNA [51, 52].

Plasma treatment also demonstrated strong efficacy for reducing ARG-associated DNA, including both intracellular and extracellular DNA fractions, consistent with previous findings [14]. The rapid disappearance of extracellular DNA within one minute of treatment suggests that plasma-generated reactive species can effectively damage DNA targets suspended in water. The observed reduction in qPCR signal likely reflects oxidative modification or fragmentation of the target gene region, preventing successful amplification during qPCR analysis. ROS may attack nucleobases, deoxyribose backbones, and phosphodiester bonds, resulting in structural disruption of the DNA molecule [53]. Intracellular DNA reduction likely occurred through a combination of bacterial inactivation, membrane disruption, and subsequent oxidative degradation of released genetic material. Importantly, extracellular ARG degradation represents a significant advantage of plasma treatment compared to conventional disinfectants, which often inactivate bacteria without fully degrading released genetic material.

Water chemistry emerged as a critical factor affecting plasma performance across all treatment targets. The reduced efficacy observed in 3.5% saltwater for rifampicin degradation, bacterial inactivation, and ARG reduction suggests that elevated conductivity substantially altered discharge characteristics and plasma chemistry. Previous studies have shown that conductive solutions can suppress stable spark formation and reduce electron energy available for reactive species production [54]. In addition, the shellfish aquaculture water required a higher electrical energy per order (EEO) for *E. coli* inactivation than distilled water (Fig. 3). This indicates that, beyond conductivity, the composition of the water matrix also influences treatment performance. Natural organic matter, proteins, suspended solids, and other dissolved constituents can scavenge plasma-generated reactive species, reducing the oxidant dose available for microbial inactivation [55]. Together, these physicochemical and biological effects highlight the importance of considering both water conductivity and matrix composition when optimizing submerged spark plasma systems for practical aquaculture and wastewater treatment applications.

### Engineering and Scale-Up Implications

In addition to demonstrating effective removal of antibiotics, ARB, and ARG-associated DNA, this study provides important insights into the engineering considerations required for practical implementation of submerged spark plasma systems in water treatment applications. Reactor configuration, power input, treatment volume, and water conductivity all strongly influenced treatment efficiency and energy consumption, highlighting the importance of process optimization for scalable deployment.

In general, increasing plasma power increases the production rate of reactive species, resulting in faster microbial inactivation, as observed in this study. However, this relationship is not necessarily linear. Beyond an optimum operating range may lead to diminishing improvements in inactivation efficiency per unit energy. The lowest electrical energy per order (EEO) for *E. coli* inactivation (1.2 kWhr/m^3^ per 1-log reduction) was achieved at moderate power input of 60 W in the multiple-spark system, indicating that excessive power may lead to diminishing returns in reactive species utilization. Increasing the power further increased the energy demand to 1.9 kWhr/m^3^ in distilled water and 2.7 kWhr/m^3^ in freshwater. In contrast, the 100 W single-spark plasma system required 50 kWh/m^3^ to achieve 1-log *E.coli* reduction. Energy evaluation was based on the first 1-log reduction to ensure consistent comparison, as higher log reductions may be influenced by tailing effects, radical depletion, and mass transfer limitations. At elevated power levels, a greater fraction of the supplied electrical energy may be dissipated through thermal losses or inefficient discharge behavior rather than productive oxidation chemistry. These findings suggest that optimization of plasma systems should focus not only on maximizing treatment rate but also on minimizing energy-normalized treatment cost. Moderate operating conditions may therefore provide the most practical balance between removal performance and energy efficiency.

The comparison between single-spark and multiple-spark reactor configurations also provides valuable insights for scale-up and reactor design. The single-spark system exhibited faster degradation kinetics and stronger local treatment intensity due to its high energy density and small treatment volume. However, the limited reactor volume may restrict throughput and scalability for practical water treatment applications. By comparison, the multiple-spark system treated substantially larger volumes while maintaining relatively strong removal performance and improved energy efficiency. Distributed discharge configurations such as the multiple-spark system may therefore provide a more feasible pathway toward pilot-scale plasma water treatment systems. Future reactor development should focus on improving reactive species distribution while maintaining sufficient local discharge intensity.

One of the most significant engineering challenges identified in this study was the reduced performance observed in saline water. The presence of dissolved salts and elevated conductivity substantially decreased rifampicin degradation, bacterial inactivation, and ARG removal efficiency. Since saline and brackish waters are commonly encountered in aquaculture systems, improving plasma performance under conductive conditions will be essential for practical implementation. Potential strategies may include optimization of electrode spacing, pulsed power modulation, alternative electrode geometries, enhanced gas injection, or hybrid plasma-oxidation systems designed to maintain stable discharge behavior in high-conductivity environments.

Compared to conventional disinfection technologies such as sodium hypochlorite, submerged spark plasma demonstrated several important advantages. Plasma treatment achieved substantially greater rifampicin degradation and more rapid extracellular DNA reduction without requiring chemical additives. In contrast, chlorination exhibited limited efficacy for rifampicin removal under the conditions evaluated in this study. Although chlorination has been reported to remove certain antibiotics such as cefotaxime (CTX), cefalexin (CLX), Ampicillin (AMP), and tetracycline (TC) [56, 57], its effectiveness varies depending on antibiotic structure and water chemistry, and the process may generate potentially harmful disinfection DBPs [58]. Previous studies have also shown that the degradation of intracellular and extracellular ARGs during chlorination is strongly dependent on chlorine dosage, with chlorine doses less than 12.5 mg/L having limited efficacy [59]. In addition, constituents commonly present in wastewater and aquaculture waters, including ammonia, nitrite, and dissolved organic nitrogen, can consume free chlorine and further reduce disinfection performance [59]. These limitations suggest that plasma-based treatment systems may offer advantages for complex water matrices where simultaneous removal of antibiotics, ARB, and ARGs is desired.

These findings are particularly relevant for aquaculture water treatment, where antibiotics, ARB, and ARGs may coexist within recirculating systems or discharge streams. Plasma-based treatment systems may offer opportunities for decentralized or on-site remediation capable of reducing antimicrobial resistance dissemination before environmental release. Potential applications include polishing treatment for recirculating aquaculture systems, treatment of concentrated waste streams, or emergency disinfection scenarios during disease outbreaks.

Further research is still needed to advance submerged spark plasma toward practical implementation. Future studies should include plasma diagnostics and reactive species quantification, identification of antibiotic degradation intermediates using LC–MS techniques, optimization of reactor hydrodynamics, evaluation of long-term electrode stability, and development of continuous-flow reactor systems. In addition, because plasma-treated water may be applied directly within recirculating aquaculture systems, comprehensive ecotoxicological evaluations should be conducted to assess the effects of residual reactive species and degradation by-products on cultured aquatic organisms, including their growth, health, and food safety. Additional investigation into plasma behavior in saline environments will also be essential for extending the applicability of submerged spark plasma to marine and brackish aquaculture systems.

## Conclusion

This study demonstrated the potential of submerged spark plasma as a multifunctional advanced oxidation and disinfection technology for the simultaneous removal of antibiotics, ARB, and ARG-associated DNA in water. Both the single-spark and multiple-spark plasma systems effectively degraded rifampicin, inactivated rifampicin-resistant *E.coli* O157:H7, and reduced intracellular and extracellular ARG-associated DNA. Plasma treatment achieved substantially greater rifampicin degradation and faster extracellular DNA reduction than sodium hypochlorite treatment under the conditions evaluated in this study.

Reactor configuration, power input, and water conductivity strongly influenced treatment performance and energy efficiency. The single-spark plasma system exhibited faster degradation kinetics due to its higher local energy density, whereas the multiple-spark system provided improved energy efficiency and greater treatment scalability. The lowest electrical energy per order (EEO) for *E. coli* inactivation was achieved at moderate power input in the multiple-spark system, indicating that increasing power does not necessarily improve energy-normalized treatment efficiency. In contrast, saline water conditions significantly reduced rifampicin degradation, bacterial inactivation, and ARG reduction, demonstrating that water conductivity is a critical factor influencing submerged plasma performance.

Overall, the findings of this study demonstrate that submerged spark plasma systems have strong potential for mitigation of antimicrobial resistance contaminants in aquaculture and wastewater treatment applications. However, additional research is needed to improve plasma performance in conductive water matrices, optimize reactor design and energy efficiency, characterize degradation pathways and reactive species generation, and develop scalable continuous-flow plasma treatment systems for practical implementation.

## Data Availability

Data will be made available on request.

## Declarations

### Generative AI and AI-assisted technologies in the writing process

During the preparation of this work the author(s) used ChatGPT in order improve the grammar and phrasing of the manuscript. After using this tool/service, the author(s) reviewed and edited the content as needed and take full responsibility for the content of the publication.
